# Curcumol inhibits the proliferation and metastasis of melanoma via the miR-152-3p/PI3K/AKT and ERK/NF-κB signaling pathways

**DOI:** 10.7150/jca.38624

**Published:** 2020-01-14

**Authors:** Ning Ning, Sulai Liu, Xiehong Liu, Zeyu Tian, Yu Jiang, Nanhui Yu, Boyu Tan, Hao Feng, Xing Feng, Lianhong Zou

**Affiliations:** 1 First Affiliated Hospital of Hunan Normal University (Hunan Provincial People's Hospital), Changsha, Hunan, China; 2 Key Laboratory of Study and Discovery of Small Targeted Molecules of Hunan Province, School of Medicine, Hunan Normal University, Changsha, Hunan, China; 3 Hunan Provincial Institute of Emergency Medicine, Hunan Provincial Key Laboratory of Emergency and Critical Care Metabonomics, Changsha, Hunan, China; 4 Department of Hepatobiliary Surgery, Hunan Provincial People's Hospital, Changsha, Hunan, China; 5 Hunan Research Center of Biliary Disease, Changsha, Hunan, China.

**Keywords:** Curcumol, Melanoma, miR-152-3p, c-MET/PI3K/AKT signaling pathway, ERK/NF-κB signaling pathway

## Abstract

Melanoma is the most aggressive and treatment-resistant form of skin cancer. Curcumol is a Chinese medicinal herb traditionally used as a cancer remedy. However, the molecular mechanisms underlying the anticancer activity of curcumol in melanoma remains largely unknown. In the present study, we observed that Curcumol decreased mouse melanoma B16 cell proliferation and migration. The xenograft tumor assay showed that curcumol reduced melanoma volume and lung metastasis. Curcumol upregulated the expression of E-cadherin and downregulated the expression of N-cadherin, MMP2 and MMP9 in mouse melanoma B16 cell. Western blot analysis revealed that curcumol reduced the translocation of p65 to the nucleus and decreased p-ERK. Furthermore, curcumol attenuated c-MET, P13K and p-AKT protein expression and upregulated miR-152-3p gene expression. The dual-luciferase reporter assay indicated that c-MET was a target gene of miR-152-3p. Reduced expression of miR-152-3p partially attenuated the effect of curcumol on mouse melanoma B16 cell proliferation and migration. The decrease in c-MET, P13K and p-AKT protein expression following curcumol treatment in mouse melanoma B16 cells was notably attenuated by the miR-152-3p inhibitor. Taken together, our findings suggested that curcumol attenuated melanoma progression and concomitantly suppressed ERK/NF-κB signaling and promoted miR-152-3p expression to inactivate the c-MET/PI3K/AKT signaling pathway.

## Introduction

Melanoma is the most prevalent, as well as the most aggressive and treatment-resistant malignant skin cancer 1,2. Melanoma causes ~80% of skin cancer-associated mortalities worldwide, and its incidence has increased by 45% since the 20th century, with an annual percentage change being estimated at 3.1% 3,4. The rising incidence and mortality of melanoma worldwide, as well as its high propensity for metastasis, highlights the urgency of exploring the molecular mechanism governing the progression of melanoma and developing effective strategies for melanoma.

**Figure 1:**
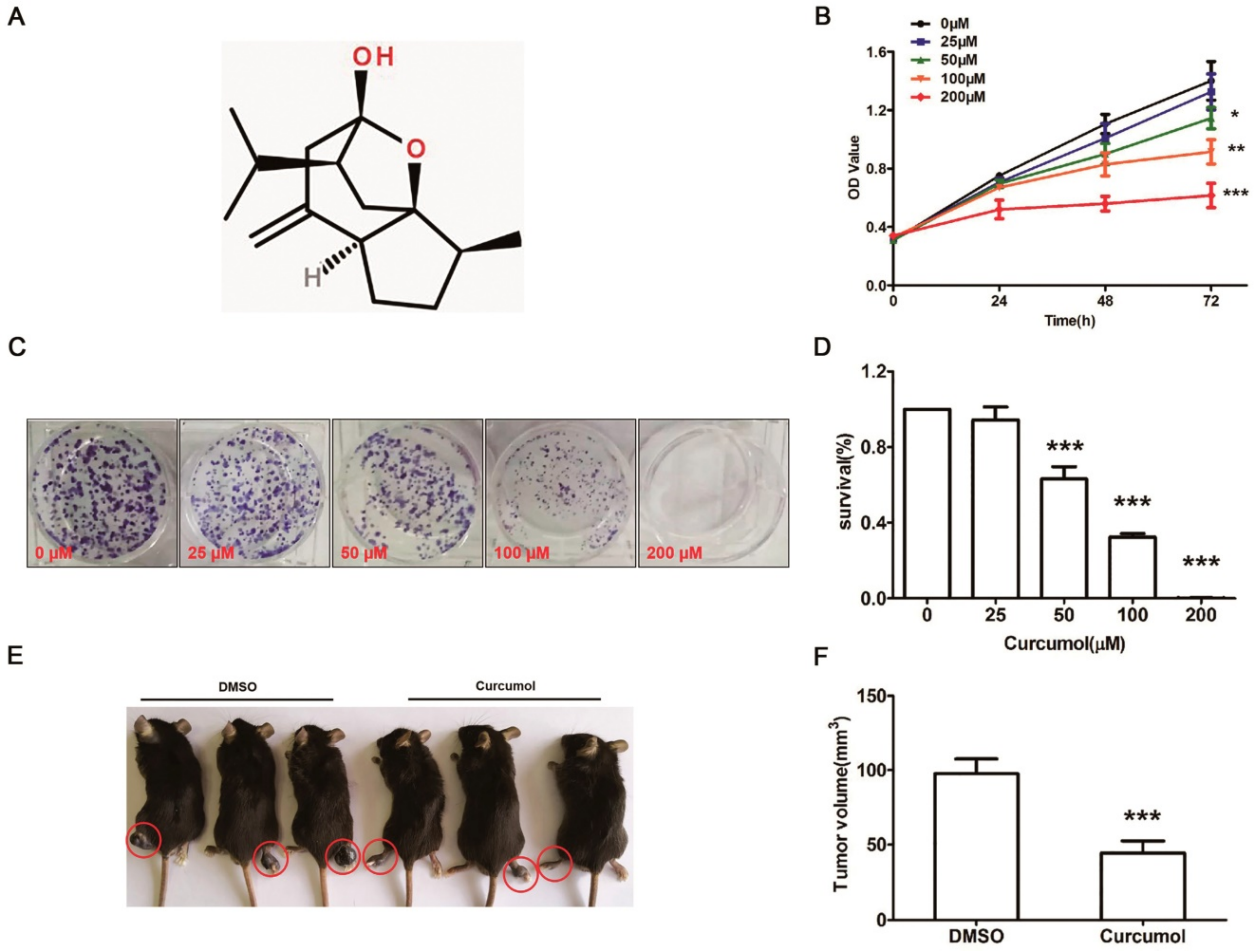
Curcumol inhibits the proliferation of mouse melanoma B16 cells in vitro and in vivo. (a) Molecular structure of curcumol. (b) Mouse melanoma B16 cells were treated with the indicated concentrations of curcumol (0, 25, 50, 100 and 200 μM) for 24, 48 and 72 h, respectively. CCK-8 assays were used to measure cell viability. (c) Mouse melanoma B16 cells were incubated with 0, 25, 50, 100 and 200 μM curcumol for 2 weeks from colonies. Then, the cells were subjected to a colony formation assay. (d) The graph summarizes the colony formation assay data. (e) The xenograft tumor of mouse melanoma B16 cells formed in C57 mice. (f) Difference in tumor volume between the DMSO group and the curcumol group. Error bars represent the s.d. of the mean. n≥6; *P<0.05;**P<0.01;***P<0.001.

Curcuma is traditionally used to treat various ailments, including cacochylia, traumatic hematoma, parasitic infection and tumorous diseases 5. C_15_H_24_O_2_-(3s-(3a,3aa,5a,6a,8ab))-octahydro-3-methyl-8-methylene-5-(1-methylethyl)-6h-3a,6-epoxyazulen-6-ol (curcumol) (Figure 1A) is a polyphenol compound isolated from the ethanol extracts of Curcuma wenyujin 6. Curcumol contains the structure of guaiane-type sesquiterpenoid hemiketal, and it is related to the potential anticancer effects of curcumol 7,8. Curcumol has been established as an antitumor compound against multiple types of cancer, including liver, gastric, and lung cancer. Curcumol exhibits anti-hepatic fibrosis activity via the downregulation of transforming growth factor-β1 and cytochrome P450 9 and the inhibition of the phosphatidylinositol 3'-kinase(PI3K) and nuclear factor kappa-B(NF-κB) pathways 10. Curcumol inhibits hepatocarcinoma cell proliferation through activating the p53 and pRB pathways 11. Curcumol decreases MMP and IDH1 to induce gastric adenocarcinoma cell apoptosis 12,13. Curcumol inhibits non-small-cell lung cancer cell migration and invasion by suppressing focal adhesion kinase(FAK) and MMP 14 and induces apoptosis via a caspase-independent mitochondrial pathway in human lung adenocarcinoma ASTC-a-1 cells 15,16. However, the molecular mechanisms underlying the anticancer activity of curcumol in melanoma have not been elucidated.

MicroRNAs (miRNAs) are a class of small, noncoding RNA molecules containing approximately 19~22 nucleotides 17. miRNAs mostly bind to the 3'-untranslated region (3'-UTR) of the target mRNAs, regulating gene expression at the posttranscriptional level 18. miR-152 is a member of the miR-148/152 family 19, which is expressed not only in normal tissues but also in numerous tumors, such as colorectal tumors 20 and breast 21 and human gastric tumors 22. miR-152 inhibits cancer stemness, cell proliferation, and tumor angiogenesis 23. Luan et al found that miR-152-3p was a target that decreased the expression of Cellular mesenchymal-epithelial transition factor (c-MET) and then suppressed melanoma progression and invasion via the PI3K/Akt/mTOR signaling pathway 24. Therefore, these studies showed that miR-152-3p could be a novel therapeutic small molecule to suppress cancer.

In this study, we assessed the effect of curcumol on mouse melanoma B16 cells. We hypothesized that curcumol suppresses the ERK/NF-κB signaling pathway and upregulates miR-152-p to suppress the c-MET/PI3K/AKT pathway and inhibit melanoma cell growth and invasion. The study elucidated the possible molecular mechanisms responsible for curcumol anticancer activity.

## Materials and Methods

### Cell culture

Mouse B16 melanoma cells were purchased from the Institute of Biochemistry and Cell Biology of the Chinese Academy of Sciences (Shanghai, China). Cells were cultured according to the manufacturer's instructions. Briefly, we cultured these cells with Dulbecco's modified Eagle's medium (DMEM). Additionally, 10% FBS and 1% penicillin/streptomycin were added (Gibco, Rockville, MD, USA) to culture melanoma cell lines. Cell incubators in a humidified atmosphere containing 5% CO_2_ were employed for all cell cultures.

### Cell viability assay

We assessed cell viability using a Cell Counting Kit-8 assay (CCK-8) following the protocol from the manufacturer (Dojindo, Tokyo, Japan). We seeded 2 × 10^3^ cells in 96-well plates and incubated at 37 °C for 24 h. The cells were treated with the indicated concentrations (0, 25, 50, 100 and 200 µM) of curcumol for 24 h, 48 h or 72 h in a humidified chamber containing 5% CO_2_. Then, we added 10 μl CCK-8 reagent into each well and incubated for 1 h at 37 °C. A microplate reader (Bio-Tech, USA) was used to determine the absorbance of cells at 450 nm (OD450).

### Colony formation assay

Mouse B16 melanoma cells were seeded into 60-mm dishes (200 cells/dish) for 24 h and then incubated with 0, 25, 50, 100 and 200 µM curcumol for 2 weeks to form colonies. Then, the colonies were washed with PBS three times. Next, the cells were fixed with methanol for 15 min, and they were stained with 0.1% crystal violet for 30 min at room temperature. Finally, we counted the number of colonies containing ≥50 cells under a microscope. Each assay was performed in triplicate.

### Xenograft tumor assay

The xenograft tumor growth assay was performed in four-week-old male C57 mice (purchased from Shanghai SLAC Laboratory Animal Co., Ltd, China), and the animal experiments were approved by the Hunan Provincial People's Hospital. First, 2 × 10^6^ mouse melanoma B16 cells were suspended in sterile saline (200 µl) or injected subcutaneously into the right lower paw and intravenously into the tail vein of mice. Curcumol (20 mg/kg) was injected intraperitoneally into mice 3 times per week. Tumor volumes were calculated according to the formula (0.5 × length × width) every 5 days. After 30 days, the mice were sacrificed, we tested the tumor volume in the right lower paw and harvested xenograft pulmonary tumors immediately.

### Wound healing assay

We used a wound healing assay to evaluate the migration ability of mouse B16 melanoma cells. Mouse B16 melanoma cells were plated at a concentration of 1×10^5^ cells/ml and incubated for 24 h, and a 200-μl pipette tip was used to scratch the cell layers to form wound gaps. The cells were incubated with 0, 50 and 100 µM curcumol for the indicated times (0, 24 and 48 h). The cells were photographed under a microscope to record the wound width. Wound healing was analyzed using ImageJ software.

### Western blot assays

Cells were washed with ice-cold PBS and RIPA buffer with proteinase inhibitor was used to extract the total protein from tissues and cells, and protein concentrations were quantified with a BCA Protein Assay Kit (Beyotime, China). Briefly, equal amounts of protein (20 µg) were separated by 10% or 12% SDS-PAGE and transferred to PVDF membranes (Millipore, USA). Subsequently, the membranes were blocked in 5% nonfat milk in TBST for 1 h at room temperature and then incubated overnight with diluted primary antibodies at 4 °C followed by incubation with HRP-conjugated secondary antibody. GAPDH was used as a control. Proteins were visualized using the Luminescent Image Analyzer (ImageQuant LAS500, GE, USA), and densitometric analysis was performed using ImageJ software.

### Transwell Migration and Invasion Assay

Cell migration was measured using a Transwell migration assay according to the manufacturer's instructions. The mouse B16 melanoma cells were mixed by the blowing method and added to the serum-free DMEM for incubator. After the cell concentration was adjusted to 5×10^5^ cells/ml, 800 µl of DMEM containing 5% FBS was added to the lower chamber of the Transwell permeable support, and 200 µl of cell suspension was added to the upper chamber. Before the experiment, 10 μg/ml collagen was added into the Transwell at 50 µl/well. The cells were then cultured with the indicated concentrations (0, 50 and 100 µM) of curcumol in an incubator for 24 h followed by fixation with 4% paraformaldehyde and staining with crystal violet for 20 min. Microscopic images were used to observe the migration and invasion of cells, and visual fields were randomly selected and photographed. ImageJ (1.48 v) software (National Institutes of Health, Bethesda, MD, USA) was used to obtain an average cell count of the four stained membrane images.

### Luciferase reporter assay

The wild-type and mutant 3'-untranslated region (3'-UTR) fragment of c-MET containing the putative binding sequences of miR-152-3p were cloned into the pGL2-basic vector (NEB) as wild-type (WT) or mutant (Mut). Mouse B16 melanoma cells were cotransfected with hsa-miR-152-3p mimic or NC and related reporter constructs. Luciferase activity was detected using the Dual Luciferase Reporter Assay System (Promega, USA) after transfection for 48 h according to the manufacturer's protocol.

### Quantitative reverse transcription-polymerase chain reaction (qRT-PCR)

We used TRIzol (Invitrogen, Carlsbad, CA, USA) to extract total RNA from mouse B16 melanoma cells according to the manufacturer's protocol. The 1 μg sample of RNA was reverse transcribed into complementary deoxyribonucleic acid (cDNA) using a PrimeScript RT Reagent Kit (RR047A; TaKaRa, Shiga, Japan). The relative miRNA-152-3P levels were determined by RT-qPCR using the StepOnePlus^TM^ Real-Time PCR System (Applied Biosystems, USA). U6 was used as the internal control. The primers were used as follows: miR-152-3p forward 5′-ACACTCCAGCTGGGTCAGTGCATGACAG-3′, and reverse, 5′-CTCAACTGGTGTCGTGGAGTCGGCAATTCAGTTGAGCCAAGTT-3′; U6 forward, 5′-CAAATTCGTGAAGCGTTCCATA-3′, and reverse, 5′-AGTGCAGGGTCCGAGGTATTC-3′. Relative quantities were determined using the comparative 2-∆∆Ct method.

### Data analysis

The results of at least three independent datasets are presented as the mean ± standard deviation (S.D.). For statistical analysis, statistical significance was analyzed using Tukey's multiple comparison tests and one-way analysis of variance. Data analysis was performed with SPSS 16.0. Statistical significance was determined at p< 0.05.

## Results

### Effect of curcumol on the proliferation of mouse melanoma B16 cells in vitro and in vivo

To determine whether curcumol influences the proliferation of mouse melanoma B16 cells, we first examined the effect of curcumol on the proliferation of the cells with the CCK-8 assay. Mouse melanoma B16 cells were treated with different concentrations of curcumol (0, 25, 50, 100 and 200 μM) for 0, 24, 48 and 72 h, respectively. As shown in Figure 1B, curcumol decreased cell viability in a dose-dependent and time-dependent manner, and compared to the control, 50- and 100-μM curcumol treatment for 48 h reduced the cell viability by 55%. Second, mouse melanoma B16 cells were incubated with 0, 50 and 100 μM for 2 weeks, and colony formation assays were performed. The results confirmed that curcumol decreased colony formation (Figure 1C and 1D) in a dose-dependent manner, and when curcumol concentration reached 100 μM, the number of colonies decreased by 60% compared to the control.

To further test the function of curcumol in vivo, we established a melanoma xenograft model by subcutaneously injecting mouse melanoma B16 cells. As shown in Figure 1E and 1F, the average tumor volume in the DMSO group was significantly greater than that in the curcumol group after 30 days. These findings suggest that curcumol suppresses the proliferation of mouse melanoma B16 cells and melanoma growth in vivo.

### Effect of curcumol on the migration and invasion of mouse melanoma B16 cells and mouse pulmonary metastasis model

**Figure 2:**
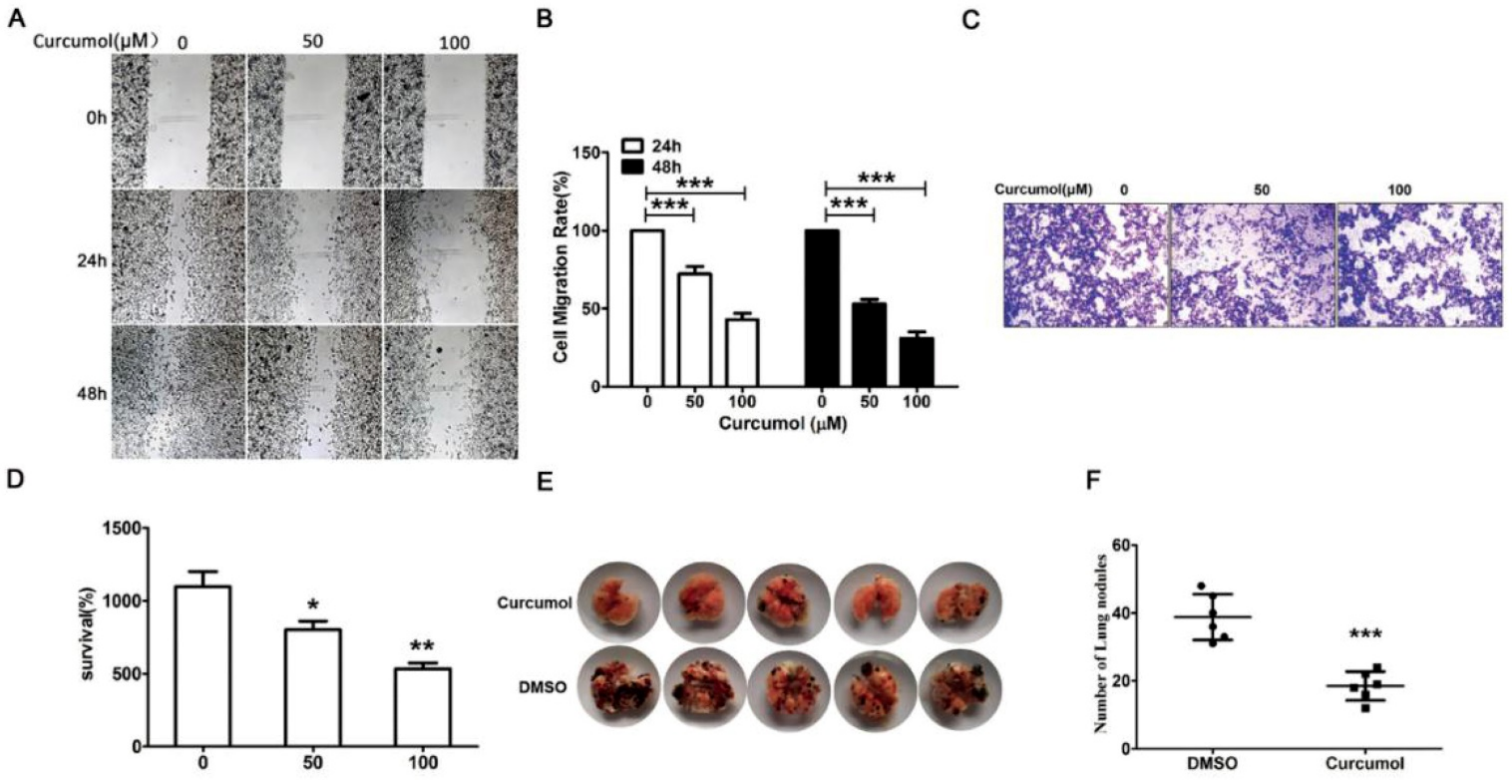
Curcumol reduces the migration and invasion in mouse melanoma B16 cells and mouse pulmonary metastasis model**.** Mouse melanoma B16 cells were treated with 50 or 100 μM curcumol or DMSO for 24 and 48 h in serum-free medium, respectively. (a) Wound healing assays were used to measure cell migration. (b) Graph of cell migration rate. (c) Mouse melanoma B16 cells were treated with 50 and 100 μM curcumol or DMSO for 48 h. Representative images of Transwell membranes stained with crystal violet (×10). Scale bar=25 μm. (d) Graph of the number of invasion cells. (e) Mouse pulmonary metastasis model. (f) Difference in the number of lung nodules between the DMSO group and the curcumol group. The results represent the average of three experiments. Error bars represent the s.d. of the mean; *P<0.05; **P<0.01; ***P<0.001.

Subsequently, we defined the role of curcumol in melanoma metastasis, and we examined the metastatic properties of the cells treated with 50 and 100 μM curcumol for 24 and 48 h in serum-free medium and examined the cells by wound-healing assay. Our data (Figure 2A and 2B) showed that there was a significantly lower migration rate for the cells in the curcumol group compared with the DMSO group. Then, we performed a migration assay to further detect the effect of curcumol on cell migration. As shown in Figure 2C and 2D, curcumol significantly inhibited cell migration in a dose-dependent manner, and 54% inhibition was observed at a concentration of 100 μM Tangeretin.

We observed that curcumol markedly suppressed cell migration in vitro and then examined the effect of curcumol on tumorigenicity and metastasis in vivo. To this end, mouse melanoma B16 cells were injected intravenously into C57 mice. As shown in Figure 2E and 2F, the intrapulmonary metastasis rate of mouse melanoma B16 cells was 80% in the DMSO group, and there was a lower 20% metastasis rate in the curcumol group. Taken together, these data support an important role for curcumol in the suppression of invasion and metastasis in vivo and in vitro.

### Curcumol modulates the expression of MMP2/9 and E/N-cadherin in mouse melanoma B16 cells

**Figure 3:**
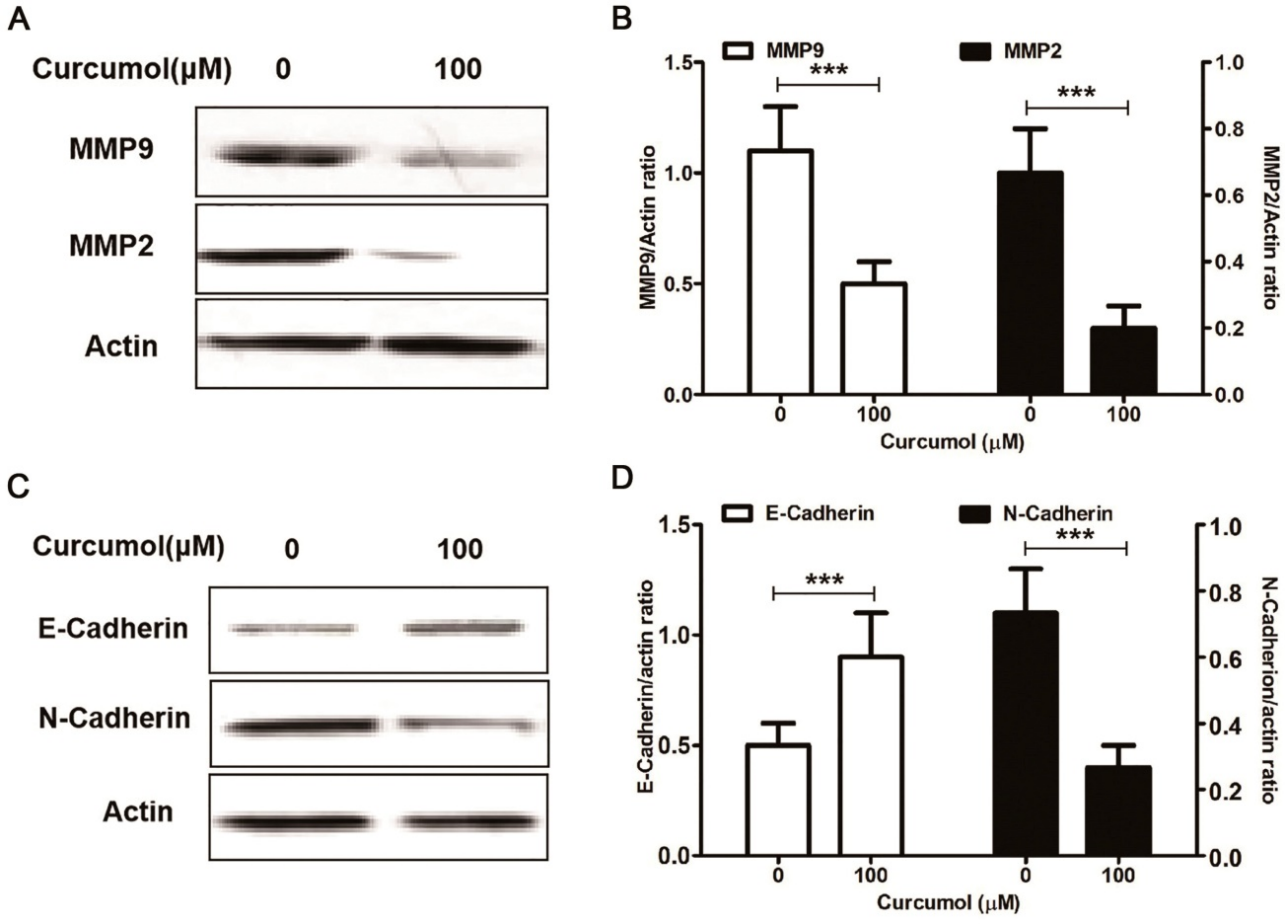
Curcumol suppresses the expression of MMP2/9 and E/N-cadherin in mouse melanoma B16 cells. Mouse melanoma B16 cells were treated with the indicated concentrations of curcumol (50 and 100 μM) or DMSO for 48 h. Then, (a) the levels of MMP2, MMP9 and actin were examined by Western blot analysis. (b) Relative levels of MMP2 and MMP9 compared with actin. (c) The levels of E-cadherin, N-cadherin and actin were examined by Western blot analysis. (d) Relative levels of E-cadherin and N-cadherin compared with actin. The results represent the average of three experiments. Error bars represent the s.d. of the mean; *P<0.05; **P<0.01; ***P<0.001.

Matrix metalloproteinase (MMPs) are the principal enzymes in extracellular matrix (ECM) degradation 25. It has been shown that elevated expression of MMP2 and MMP9 is positively correlated with tumor progression, metastasis, and poor prognosis 26. Curcumol has been shown to decrease the MMP to inhibit lung adenocarcinoma cancer cell migration and invasion 12,13. Furthermore, our results indicated that curcumol decreased the expression of MMP2 and MMP9 in mouse melanoma B16 cells (Figure 3A and 3B).

Epithelial to mesenchymal transition (EMT) refers to the loss of cell adhesion, repression of E-cadherin expression and increasing cell mobility 27. The aberrant activation of EMT promotes tumor cell invasion and dissemination 28. Yan et al found that curcumol altered the levels of E-cadherin and N-cadherin in a dose-dependent manner in a nasopharyngeal carcinoma cell xenograft model 29. Our results indicated that curcumol attenuated the expression of MMP2 and MMP9; therefore, we sought to determine whether curcumol was responsible for regulating epithelial-mesenchymal transition(EMT) in mouse melanoma B16 cells. Our data showed that curcumol increased the expression of E-cadherin and prominently decreased the expression of N-cadherin (Figure 3C and 3D). Taken together, these data clearly demonstrate that curcumol limited the metastatic process of mouse melanoma B16 cells by regulating the ECM and EMT.

### Effect of curcumol on the PI3K/Akt and ERK/NF-κB pathways in mouse melanoma B16 cells

**Figure 4:**
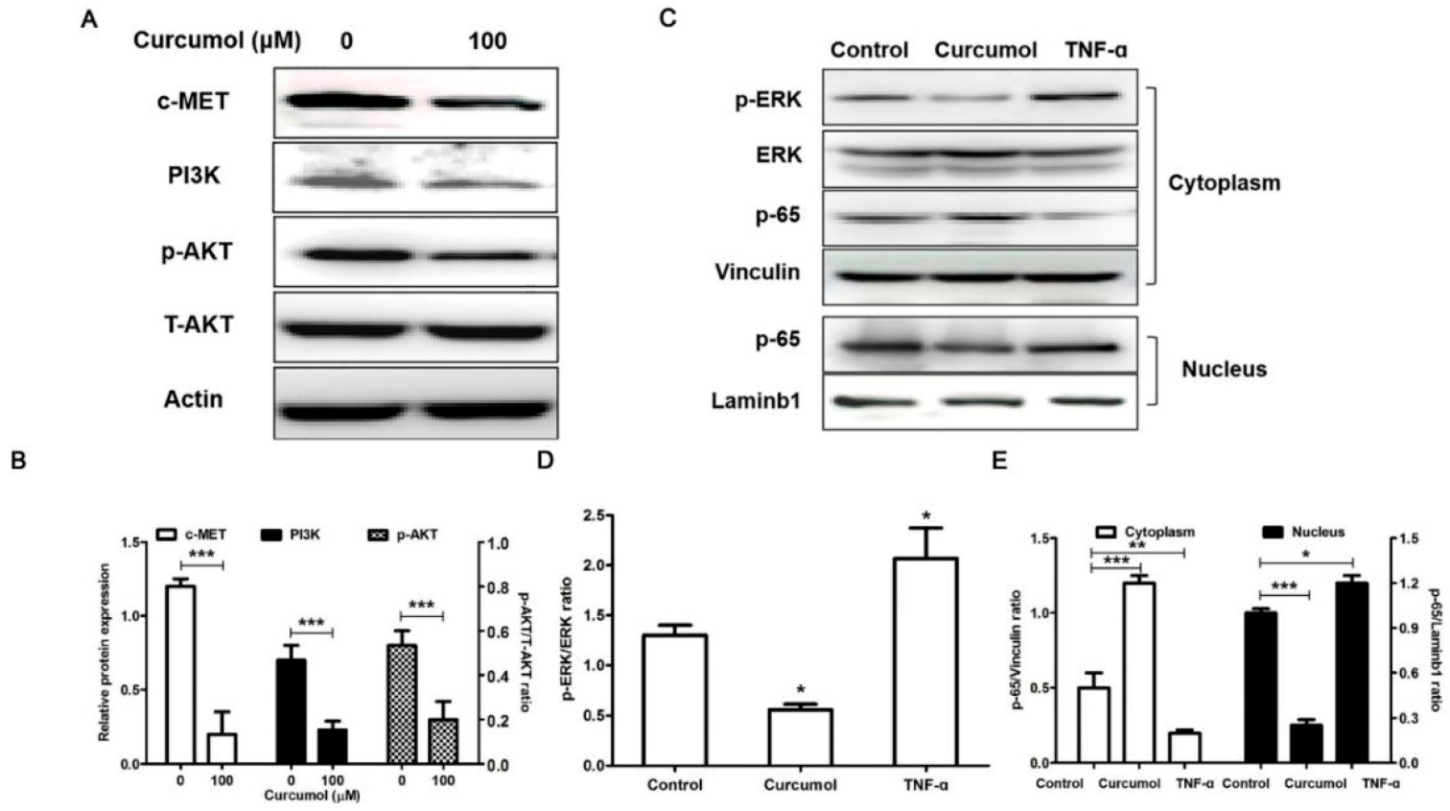
PI3K/Akt and ERK/NF-κB pathways are related to the inhibition of melanoma induced by curcumol. (a) Mouse melanoma B16 cells were treated with 100 μM curcumol or DMSO for 48 h. Then, the levels of c-MET, PI3K, p-AKT, T-AKT and actin were examined by Western blot analysis. (b) Densitometry revealed the fold expression of c-MET and P13K compared with that of actin, respectively; and densitometry revealed the fold expression of p-AKT compared with that of T-AKT. (c) Mouse melanoma B16 cells were treated with 100 μM curcumol for 48 h or 10 ng/ml TNF-α for 1 h. After this step, cytoplasmic (Cyto) or nuclear (Nuc) extracts were analyzed using antibodies against the indicated proteins. (d) Graph represents the ratio of p-ERK/ERK. (e) Graph represents the levels of p65 Cyto and p65 Nuc relative to the control. The results represent the average of three experiments. Error bars represent the s.d. of the mean; *P<0.05; **P<0.01; ***P<0.001.

Cellular c-MET acts as an oncogene in malignant melanoma, and c-MET expression is markedly increased in melanoma cells 24. It has been demonstrated that c-MET enhanced melanoma cell proliferation and invasive capacity and protected cells from apoptosis via the PI3K/AKT signaling pathway 24,30. Thus, based on our results showing that curcumol affected mouse melanoma B16 cell proliferation and invasion, we next investigated the effect of curcumol on c-MET, PI3K and Akt protein expression. Western blot analysis revealed that the c-MET and PI3K protein levels in the curcumol group were significantly lower than those in the control group. Additionally, curcumol attenuated Akt phosphorylation in mouse melanoma B16 cells (Figure 4A and 4B).

Numerous studies have shown that MMP2/9 expression is regulated by NF-κB signaling pathways in melanoma 31-33, and curcumol induced HSC-T6 cell death via this pathway. Therefore, we further observed the effects of curcumol on the NF-κB pathway in mouse melanoma B16 cells. We applied tumor necrosis factor alpha (TNF-α) as a positive control in experiments. Our results demonstrate that curcumol substantially decreased the phosphorylation of ERK and activation of p65 compared with the control group (Figure 4C-E). Overall, these data indicate that curcumol mediates the inhibition of the PI3K/Akt and NF-κB signaling pathways to modulate migration and invasion in mouse melanoma B16 cells.

### Curcumol inhibited mouse melanoma B16 cell growth and metastasis by upregulating miR-152-3p

**Figure 5:**
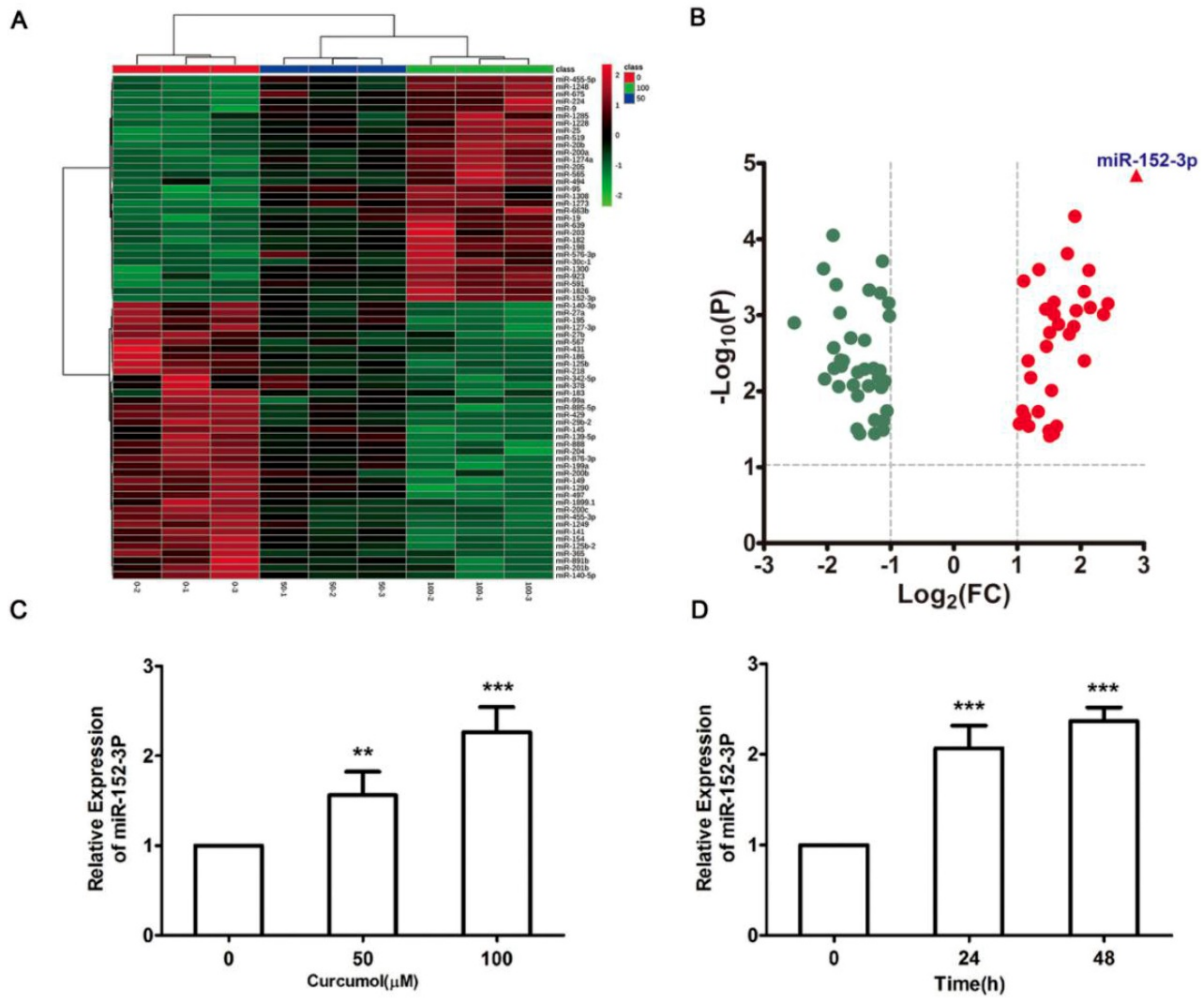
Effect of curcumol on miR-152-p expression in mouse melanoma B16 cells. (a) Mouse melanoma B16 cells were treated with 50 and 100 μM curcumol or DMSO for 48 h. Heat map of miR related gene expression of curcumol or DMSO treated cells. The blanket in the right is the related gene symbol of the hot map. (b) Volcano plot analysis of miR gene in 100 μM curcumol group compare with the DMSO group. The dots represent genes which passed our filtering criteria (P < 0.05 and fold change > 1.2). X axis and Y axis represent log2(fold change) and -log10(P value), respectively. (c) Mouse melanoma B16 cells were treated with 50 and 100 μM curcumol or DMSO for 48 h. (d) Mouse melanoma B16 cells were treated with 100 μM curcumol or DMSO for 0, 24 and 48 h, respectively. miR-152-p was measured by real-time quantitative PCR. Error bars represent the s.d. of the mean; *P<0.05; **P <0.01; ***P<0.001.

miR-152-3p is a tumor suppressor gene that is expressed at low levels in melanoma 20-24. Therefore, we performed miRNA microarray analysis to determine whether curcumol affects miRNA expression in mouse melanoma B16 cells. The heat map data revealed 69 differentially expressed miRNAs in the 100 μM curcumol group compared with the DMSO group (Figure 5A). Treatment with 100 μM curcumol upregulated 38 miRNAs and downregulated 31 miRNAs. Notably, 100 μM curcumol significantly upregulated miR-152-3p expression, which was greater than a seven-fold change compared with the DMSO group (Table 1 and Figure 5B). To further confirm the data obtained from the miRNA microarray, we used qRT-PCR to examine miR-152-3p expression in mouse melanoma B16 cells. Our results showed that curcumol upregulated the level of miR-152-3p in a dose-dependent and time-dependent manner (Figure 5C and 5D).

**Figure 6:**
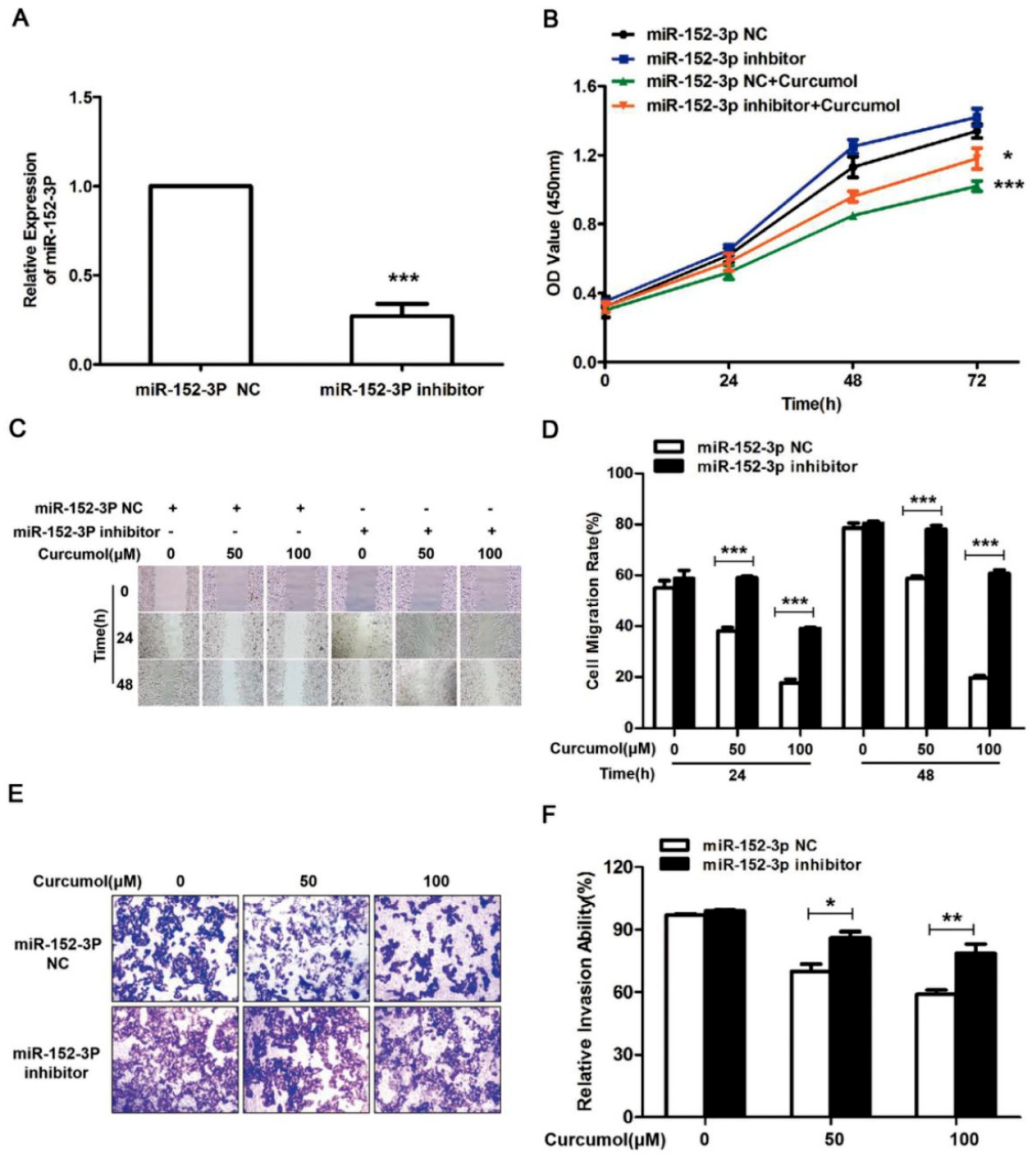
Curcumol-enhanced miR-152-3p expression is involved in mouse melanoma B16 cell growth and metastasis. (a) Mouse melanoma B16 cells were transfected with miR-152-3p NC or miR-152-3p inhibitor for 48 h. Graphs represent the relative mRNA expression of miR-152-3p in mouse melanoma B16 cells compared with the control. (b) Mouse melanoma B16 cells were transfected with miR-152-3p NC or miR-152-3p inhibitor for 48 h and then treated with 100 μM curcumol or DMSO for 48 h. CCK8 was quantified at 5, 10, 15, 20, 30, and 60 min at 450 nm using a microplate reader. (c) Mouse melanoma B16 cells were transfected with miR-152-3p NC or miR-152-3p inhibitor for 48 h and then treated with 50 or 100 μM curcumol for 24 or 48 h. Wound healing assays were used to measure cell migration. (d) Graph of cell migration rate. (e) Mouse melanoma B16 cells were transfected with miR-152-3p NC or miR-152-3p inhibitor for 48 h and then treated with 50 or 100 μM curcumol for 48 h. Representative images of Transwell membranes stained with crystal violet (×10). Scale bar=25 μm. (f) Graph of the number of invasion cells. Error bars represent the s.d. of the mean; *P<0.05;**P<0.01; ***P<0.001.

The results above clearly indicate that curcumol increases the level of miR-152-3p in mouse melanoma B16 cells. Subsequently, we used siRNA technology to inhibit miR-152-3p expression and investigated whether curcumol prevented mouse melanoma B16 cell proliferation and migration via miR-152-3p. Treatment with siRNA for miR-152-3p downregulated miR-152-3p expression by 85% (Figure 6A), and curcumol led to a reduction in mouse melanoma B16 cell viability, which was significantly reversed by the addition of miR-152-3p inhibitor (Figure 6B). As shown (Figure 6C-F), 100 μM curcumol reduced the migration rate of the mouse melanoma B16 cells and was markedly attenuated by the miR-152-3p inhibitor. Taken together, our data suggest that curcumol prevents mouse melanoma B16 cell proliferation and migration by upregulating the level of miR-152-3p.

### Curcumol-mediated suppression of the PI3K/AKT signaling pathway partially depends on miR-152-3p

**Figure 7:**
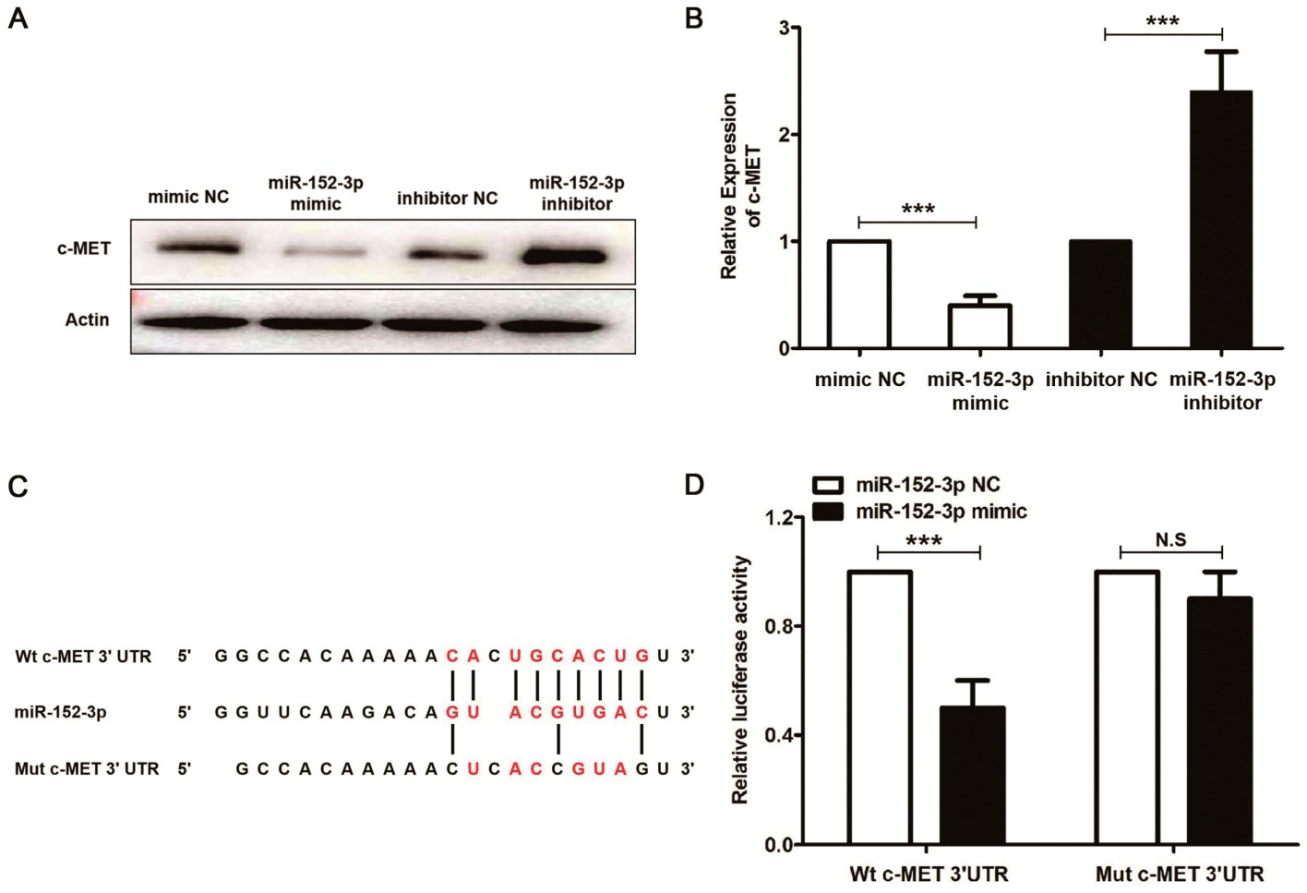
miR-152-3p represses the expression of c-MET. Mouse melanoma B16 cells were transfected with miR-152-3p NC or miR-152-3p inhibitor for 48 h, and then treated with 100 μM curcumol or DMSO for 48 h. (a) c-MET was measured by real-time quantitative PCR. (b) c-MET protein expression was measured by Western blot. (c) Predicted miRNA binding sites within the 3`-UTR of c-MET mRNA by Starbase 2.0. The pairing between miR-152-3p and the putative binding sites in the 3`-UTR of c-MET mRNA are shown. (d) Luciferase activity was measured in mouse melanoma B16 cells transfected with the wild-type 3`-UTR and mutant 3`-UTR c-MET luciferase constructs and a miR-152-3p mimic or a scrambled miRNA control. The results represent the average of three experiments. Error bars represent the s.d. of the mean;*P<0.05; **P<0.01; ***P<0.001.

miR-152-3p, through direct targeting of c-MET, suppresses oral squamous cell carcinoma 34, and our results also demonstrated that miR-152-3p inhibitor reduced the c-MET gene transcription and protein expression (Figure 7A and 7B). We predicted the potential miR-152-3p binding sites in c-MET using Starbase 2.0 (http://www.targetscan.org/vert_71/). Dual luciferase reporter assays showed that a miR-152-3p mimic reduced the luciferase activity of pMIR-c-MET-WT but not that of pMIR-c-MET-MUT in mouse melanoma B16 cells (Figure 7C and 7D).

**Figure 8:**
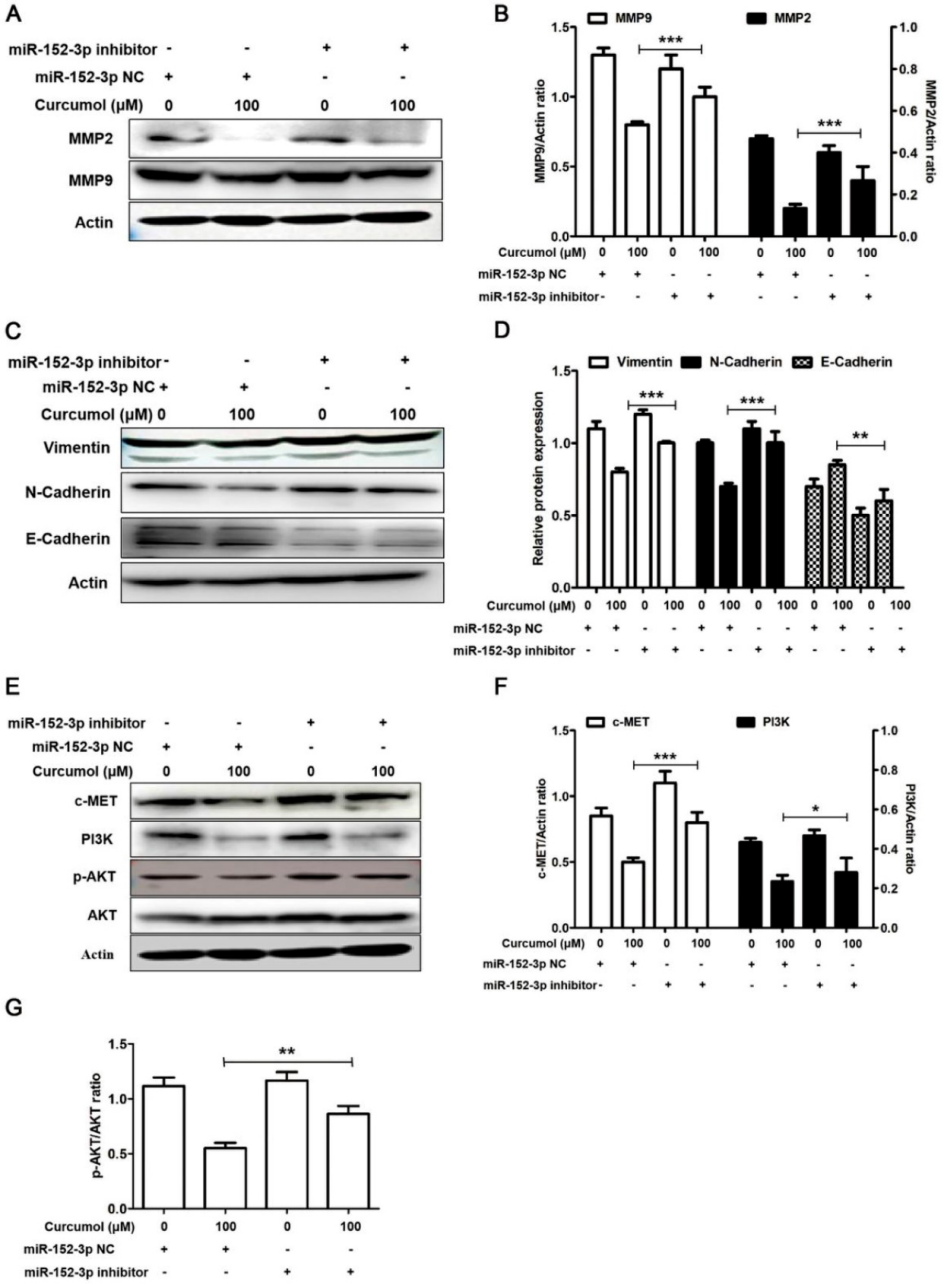
Curcumol inhibits the PI3K/Akt signaling pathway by upholding miR-152-3p. Mouse melanoma B16 cells were transfected with miR-152-3p NC or miR-152-3p inhibitor for 48 h and then treated with 100 μM curcumol or DMSO for 48 h. Then, (a) Western blot analysis was used to measure the protein expression of MMP2, MMP9 and (c) N-cadherin, E-cadherin and (e) c-MET, PI3K, p-AKT and AKT. (b) Relative levels of MMP2 and MMP9 compared with actin. (d) Relative levels of Vimentin, E-cadherin and N-cadherin compared with actin. (f) Graph represents the ratio of cMET (or P13K) and actin. (g) Graph represents the ratio of p-AKT/AKT. The results represent the average of three experiments. Error bars represent the s.d. of the mean; *P<0.05; **P<0.01; ***P<0.001.

Based on our results, we used a miR-152-3p inhibitor to further examine the effects of curcumol on MMP2/9 and E/N-cadherin in mouse melanoma B16 cells. As demonstrated in Figure 8A and 8B, the decrease in MMP2 and MMP9 following curcumol-treated mouse melanoma B16 cells was markedly attenuated by the miR-152-3p inhibitor. Additionally, the miR-152-3p inhibitor reversed the curcumol effect by decreasing N-cadherin and increasing E-cadherin (Figure 8C and 8D). These results indicated that curcumol regulated ECM and EMT by miR-152-3p. In addition, the expression of c-MET and PI3K and the phosphorylated form of AKT were considerably upregulated in the curcumol treatment group. In contrast, the miR-152-3p inhibitor led to a marked decrease in c-MET, PI3K and p-AKT levels, and the miR-152-3p inhibitor attenuated the effect of curcumol (Figure 8E-H). In summary, curcumol inhibited mouse melanoma proliferation and migration, largely by enhancing miR-152-3p and downregulating the c-MET/PI3K/AKT pathway.

## Discussion

**Figure 9:**
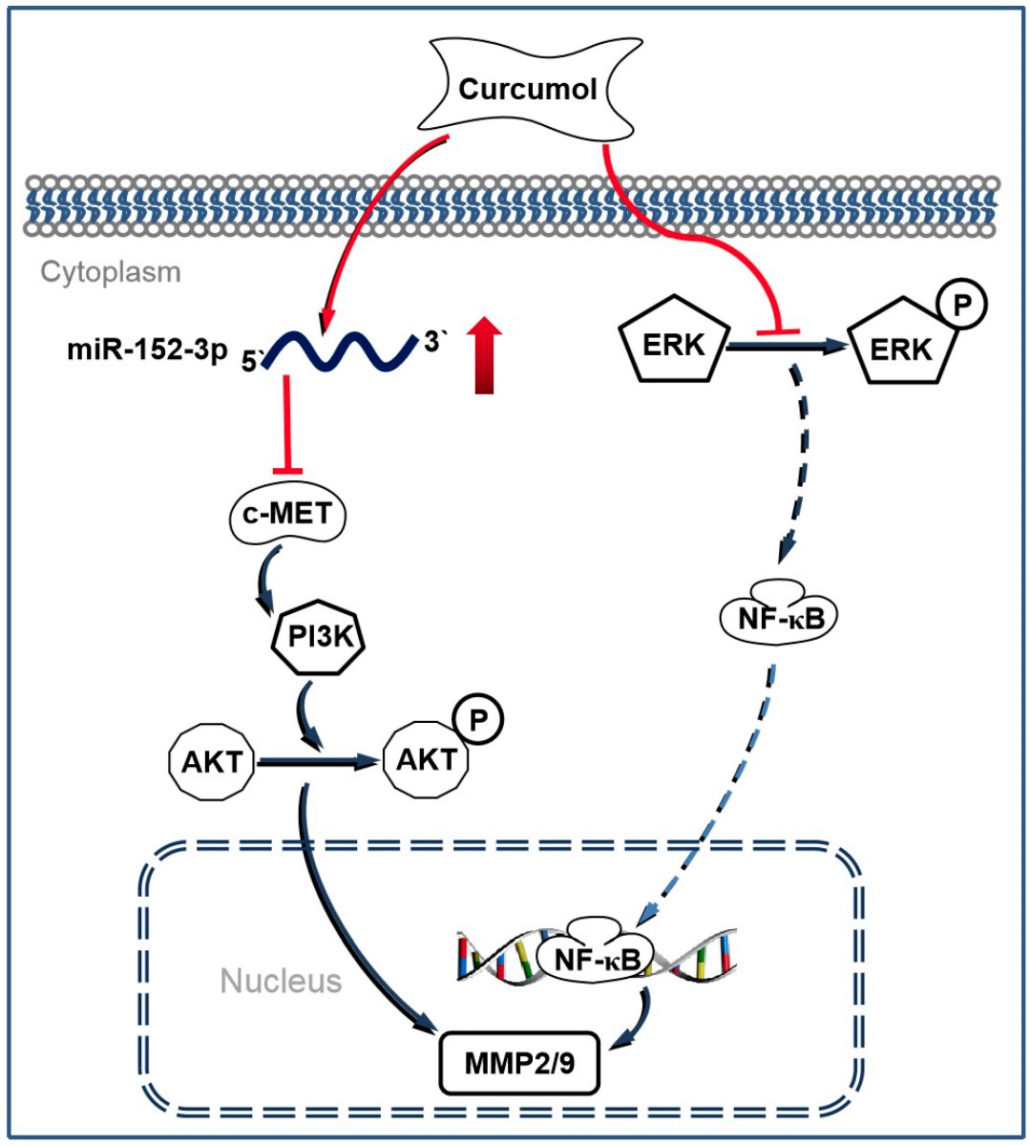
Schematic illustrating the working principle of curcumol related signaling in mouse melanoma B16 cells. We propose that curcumol maintained the level of miR-152-p in mouse melanoma B16 cells, thereby inactivating c-MET/PI3K/AKT-dependent signaling pathway associated with mouse melanoma B16 cell death. In addition, our results also imply that ERK/NF-κB signaling pathway contributes partially in curcumol-mediated effects.

Melanoma is considered to be one of the most malignant cancers, and approximately 200,000 new cases are diagnosed worldwide each year 35. Currently, the major challenges to conventional antitumor drugs include chemotherapy resistance and severe systemic adverse effects 36,37. Therefore, interest has increasingly focused on finding novel and alternative medicines that are safe and effective against melanoma. Curcumol has been reported to possess antitumor and antiviral activities with low cytotoxicity 38. In the present study, we demonstrated that curcumol decreased the expression of c-MET, PI3K and p-AKT by increasing the level of miR-152-3p. Simultaneously, curcumol decreased ERK phosphorylation and prevented the activity of NF-κB, thereby driving the c-MET/PI3K/AKT-dependent and ERK/NF-κB-dependent pathways associated with mouse melanoma B16 cell death (Figure 9).

Curcumol is one of the major monomeric components of Rhizoma Curcumae and is widely used in the treatment of cancer in China 39. Curcumol has a wide range of biological activities, including proliferation inhibition and apoptosis induction of a variety of cancer cells 9-16. Consistent with the aforementioned reports, we found that curcumol decreased mouse melanoma B16 cell proliferation and migration. Curcumol is a multitarget natural anticancer substance, and the main targets involved in different cancers are also different, but the PTEN/P13K/AKT pathway and the ERK/NF-κB pathway are the main regulatory points for curcumol to inhibit cancer cell growth. Liu et al found that curcumol inhibited colorectal cancer proliferation via modulating PTEN/PI3K/AKT pathways 40. Li et al indicated that curcumol induces cell cycle arrest and apoptosis by inhibiting the IGF-1R/PI3K/Akt signaling pathway in human nasopharyngeal carcinoma CNE-2 cells 41. Our current findings also revealed that the inhibitory effect of curcumol on the proliferation and migration of mouse melanoma B16 cells is related to the inhibition of the PI3K/AKT pathway. Consistent with previous reports 9,31-33,42, our results also showed that curcumol exerted an inhibitory effect on mouse melanoma B16 cells by inhibiting the ERK/NF-κB signaling pathway.

Inhibition of the c-MET pathway is a potential therapeutic strategy against melanoma 24,30,43,44. Overexpression of c-MET in melanoma cells enhances cell protection from cell death and correlates with a poor clinical outcome 45,46, which was shown to be mediated by the activation of the PI3K/AKT pathways 47,48. Furthermore, some synthetic cyclic compounds were designed and used to suppress MET kinase activity, such as cabozantinib, foretinib, BMS777607, MGCD-265 and pyrrolo[23-b]pyridine derivatives 49. Our data also showed that curcumol decreased the expression of c-MET, which inhibited downstream PI3K/AKT pathways.

A number of studies have revealed that miRNA plays a critical role in health and diseases, including cell growth, migration, apoptosis and cancer cell resistance 50-52. A decrease in miR-152 has been proved to be associated with the processes of cell proliferation, invasion and angiogenesis in different neoplasms 53-55. In melanoma, miR-152-3p levels are relatively decreased compared to normal mammary tissues 24. Then, we used miRNA microarrays to test the potential miRNAs that could be regulated by curcumol. miR-152-3p expression was significantly higher in the curcumol treatment group in mouse melanoma B16 cells, which was consistent with the qRT-PCR results. In addition, c-MET plays an essential role in miR-152-regulated PI3K/AKT signaling in osteosarcoma cells 56. Based on these findings, we hypothesized that curcumol exerts anti-tumor activity, which may be associated with an increase in the level of miR-152-3p to decrease the c-MET in mouse melanoma B16 cells. To test this hypothesis, miR-152-3p that could interact with c-MET was predicted using Starbase 2.0. Western blotting and qRT-PCR results indicated that miR-152-3p reduced the level of c-MET. Moreover, dual luciferase reporter assays confirmed that miR-152-3p directly binds to c-MET. Finally, we found that curcumol inhibits c-MET expression and the downstream signaling pathway PI3k/Akt, and this inhibition effect of curcumol on mouse melanoma B16 cells can be reversed by miR-152-3p inhibitor. These results reveal that curcumol suppressed melanoma cell growth and metastasis by, at least in part, upregulating the level of miR-152-3p and promoting miR-152-3p to bind c-MET.

To summarize, this study is the first to demonstrate that curcumol, via the c-MET/PI3K/AKT and ERK/NF-κB signaling pathways, deceases melanoma cell proliferation and migration. Moreover, our research has demonstrated a potential role for curcumol in the treatment of melanoma, and miR-152-3p was targeted by curcumol to treat melanoma. However, due to the unique five-membered and seven-membered ring chemical structure of curcumol, the drug solubility is poor, and the bioavailability is not high. Therefore, further studies are warranted to improve the physical properties and modify the structure of curcumol in a clinical context.

## Figures and Tables

**Table 1:**
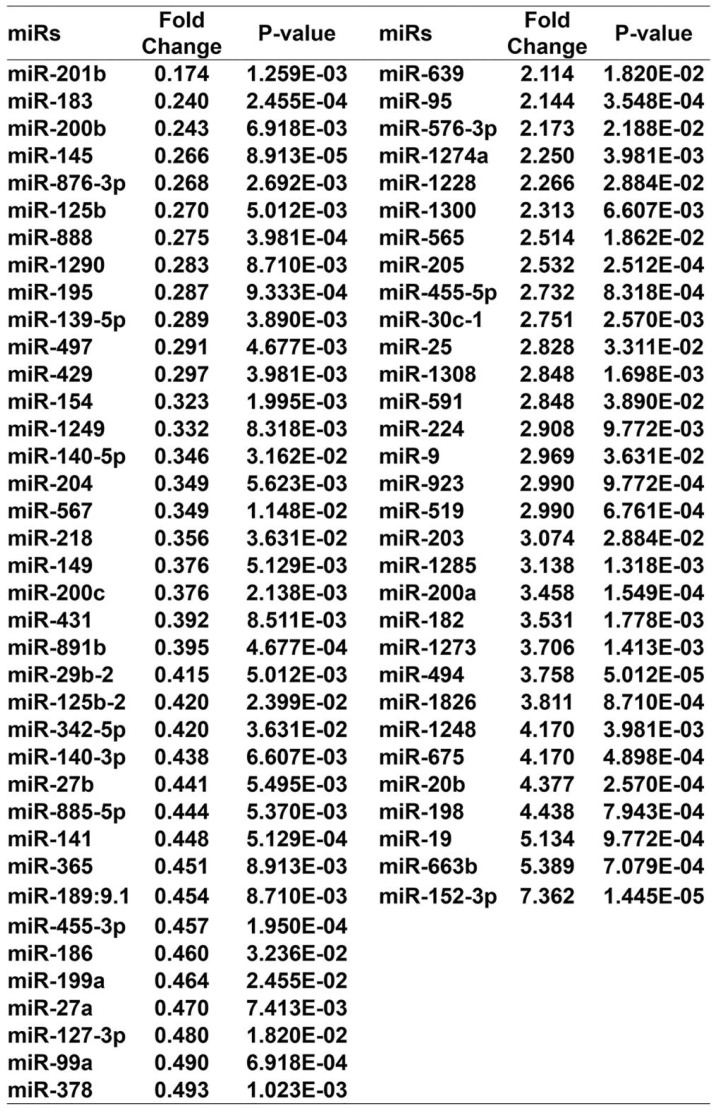
miR gene change in the 100 μM curcumol group compared with the DMSO group.

## Abbreviations

Curcumol: C15H24O2-(3s-(3a,3aa,5a,6a,8ab))-octahydro-3-methyl-8-methylene-5-(1-methylethyl)-6h-3a,6-epoxyazulen-6-ol
MMP9: matrix metalloprotein-9
MMP2: matrix metalloprotein-2
ECM: extracellular matrix
EMT: epithelial-mesenchymal transition
c-MET: Cellular mesenchymal-epithelial transition factor
PI3K: phosphatidylinositol 3'-kinase
AKT: proteinkinase B
ERK: extracellular regulated protein kinases
NF-κB: nuclear factor kappa-B
FAK: focal adhesion kinase
MiRNAs: MicroRNAs
TNF-α: tumor necrosis factor alpha
SPINT2: serine peptidase inhibitor kunitz type 2
